# Karyotype variation patterns and phenotypic responses of hybrid progenies of triploid loquat (*Eriobotrya japonica*) provide new insight into aneuploid germplasm innovation

**DOI:** 10.1093/hr/uhaf023

**Published:** 2025-01-22

**Authors:** Peng Wang, Shangjian Yang, Meiyi Chen, Yingjia Liu, Qiao He, Haiyan Sun, Di Wu, Suqiong Xiang, Danlong Jing, Shuming Wang, Qigao Guo, Jiangbo Dang, Guolu Liang

**Affiliations:** Key Laboratory of Agricultural Biosafety and Green Production of Upper Yangtze River (Ministry of Education), College of Horticulture and Landscape Architecture, Southwest University, Beibei, Chongqing 400715, China; Academy of Agricultural Sciences of Southwest University, State Cultivation Base of Crop Stress Biology for Southern Mountainous Land of Southwest University, Beibei, Chongqing 400715, China; Key Laboratory of Agricultural Biosafety and Green Production of Upper Yangtze River (Ministry of Education), College of Horticulture and Landscape Architecture, Southwest University, Beibei, Chongqing 400715, China; Academy of Agricultural Sciences of Southwest University, State Cultivation Base of Crop Stress Biology for Southern Mountainous Land of Southwest University, Beibei, Chongqing 400715, China; Key Laboratory of Agricultural Biosafety and Green Production of Upper Yangtze River (Ministry of Education), College of Horticulture and Landscape Architecture, Southwest University, Beibei, Chongqing 400715, China; Academy of Agricultural Sciences of Southwest University, State Cultivation Base of Crop Stress Biology for Southern Mountainous Land of Southwest University, Beibei, Chongqing 400715, China; Key Laboratory of Agricultural Biosafety and Green Production of Upper Yangtze River (Ministry of Education), College of Horticulture and Landscape Architecture, Southwest University, Beibei, Chongqing 400715, China; Academy of Agricultural Sciences of Southwest University, State Cultivation Base of Crop Stress Biology for Southern Mountainous Land of Southwest University, Beibei, Chongqing 400715, China; Key Laboratory of Agricultural Biosafety and Green Production of Upper Yangtze River (Ministry of Education), College of Horticulture and Landscape Architecture, Southwest University, Beibei, Chongqing 400715, China; Academy of Agricultural Sciences of Southwest University, State Cultivation Base of Crop Stress Biology for Southern Mountainous Land of Southwest University, Beibei, Chongqing 400715, China; Key Laboratory of Agricultural Biosafety and Green Production of Upper Yangtze River (Ministry of Education), College of Horticulture and Landscape Architecture, Southwest University, Beibei, Chongqing 400715, China; Academy of Agricultural Sciences of Southwest University, State Cultivation Base of Crop Stress Biology for Southern Mountainous Land of Southwest University, Beibei, Chongqing 400715, China; Key Laboratory of Agricultural Biosafety and Green Production of Upper Yangtze River (Ministry of Education), College of Horticulture and Landscape Architecture, Southwest University, Beibei, Chongqing 400715, China; Academy of Agricultural Sciences of Southwest University, State Cultivation Base of Crop Stress Biology for Southern Mountainous Land of Southwest University, Beibei, Chongqing 400715, China; Key Laboratory of Agricultural Biosafety and Green Production of Upper Yangtze River (Ministry of Education), College of Horticulture and Landscape Architecture, Southwest University, Beibei, Chongqing 400715, China; Academy of Agricultural Sciences of Southwest University, State Cultivation Base of Crop Stress Biology for Southern Mountainous Land of Southwest University, Beibei, Chongqing 400715, China; Key Laboratory of Agricultural Biosafety and Green Production of Upper Yangtze River (Ministry of Education), College of Horticulture and Landscape Architecture, Southwest University, Beibei, Chongqing 400715, China; Academy of Agricultural Sciences of Southwest University, State Cultivation Base of Crop Stress Biology for Southern Mountainous Land of Southwest University, Beibei, Chongqing 400715, China; Key Laboratory of Agricultural Biosafety and Green Production of Upper Yangtze River (Ministry of Education), College of Horticulture and Landscape Architecture, Southwest University, Beibei, Chongqing 400715, China; Academy of Agricultural Sciences of Southwest University, State Cultivation Base of Crop Stress Biology for Southern Mountainous Land of Southwest University, Beibei, Chongqing 400715, China; Key Laboratory of Agricultural Biosafety and Green Production of Upper Yangtze River (Ministry of Education), College of Horticulture and Landscape Architecture, Southwest University, Beibei, Chongqing 400715, China; Academy of Agricultural Sciences of Southwest University, State Cultivation Base of Crop Stress Biology for Southern Mountainous Land of Southwest University, Beibei, Chongqing 400715, China; Key Laboratory of Agricultural Biosafety and Green Production of Upper Yangtze River (Ministry of Education), College of Horticulture and Landscape Architecture, Southwest University, Beibei, Chongqing 400715, China; Academy of Agricultural Sciences of Southwest University, State Cultivation Base of Crop Stress Biology for Southern Mountainous Land of Southwest University, Beibei, Chongqing 400715, China; Key Laboratory of Agricultural Biosafety and Green Production of Upper Yangtze River (Ministry of Education), College of Horticulture and Landscape Architecture, Southwest University, Beibei, Chongqing 400715, China; Academy of Agricultural Sciences of Southwest University, State Cultivation Base of Crop Stress Biology for Southern Mountainous Land of Southwest University, Beibei, Chongqing 400715, China

## Abstract

The sexual reproduction of triploids induces chromosomal karyotype variations, which are significant for germplasm resource innovation. Most triploid plants are with low fertility. Therefore, triploid offspring karyotypes’ variation pattern and phenotypic response remain poorly understood. Here, we employed three diploids with diverse genetic distances as male parents to cross-pollinate the female fertile triploid loquat Q24 to construct three experimental populations. The chromosome numbers of 93.82% of hybrid plants were 34~46 in three hybrid populations. All 168 aneuploids with 160 karyotypes and a small percentage of euploids were detected among 178 hybrids by the improved molecular karyotype analysis method. Further analysis revealed that when being transmitted to offspring, chromosome 5 of Q24 as disomy had the highest frequency (>50%), while chromosome 12 had the lowest frequency (≤30%). The frequency of Q24’s chromosomes being transmitted to offspring as disomy was influenced by the gene function on the chromosomes and the number of interchromosome collinear gene links. Whole-genome resequencing showed that the Q24 alleles exhibited segregation distortions in the offspring aneuploid population. Transgenic experiments demonstrated that *the EjRUN1* gene, which was on one segregation distortion region of Q24, promoted the seed viability of triploid *Arabidopsis*. Furthermore, chromosome number, dosage, and male parent genotype affected the aneuploid phenotype. These findings advance the understanding of genome genetic characteristics of triploid loquat, and provide a reference for germplasm innovation of loquat rapidly through triploid sexual reproduction.

## Introduction

Triploids are highly valued in horticultural plant breeding due to their notable characteristics, including reduced seed production, high yields, and strong resistance [1]. Additionally, triploids play an essential role in enhancing plant diversity because of the significant genomic variation that occurs during meiosis. Some euploid gametes produced by triploids can aid in the formation of polyploids, establishing triploids as a critical bridge in the process of plant polyploidy [2–4]. Furthermore, studies have shown that triploids can produce aneuploid offspring in various species, including *Arabidopsis* [5], *Malus domestica* Borkh [6], *Musa* spp. [7], *Pyrus* sp. [8], and *Eriobotrya japonica* [9]. This indicates that the sexual reproduction of triploids can rapidly induce variations in chromosomal karyotypes.

Conceivably, chromosomal karyotype variation alters the dose and expression of a large number of genes relative to gene mutations, so aneuploidy generally exhibits more severe phenotypic consequences than euploidy. In 1920, this phenomenon was reported for the first time in *Datura stramonium*, in which aneuploid plants exhibited phenotypes different from those of normal diploid plants [10]. Many subsequent reports have indicated the universality of this phenomenon, such as the presence of aneuploidy in *Brassica* [2], *Arabidopsis thaliana* [5], *Oryza* [11], *Gossypium hirsutum* L. [12], *Triticum aestivum* L. [13], and *Lilium* [14]. Aneuploidy is characterized by many novel phenotypes that are highly important for germplasm innovation [5, 10, 11]. In particular, in some plants with narrow genetic backgrounds, aneuploids can be used to increase genetic diversity in a short time; even new varieties with novel traits are bred. However, aneuploidy cannot be applied in the production of some crops because of genetic instability. Many horticultural plants, like fruit trees, can reproduce through asexual pathways. Thus, aneuploids can be used in horticultural plants because of the asexual reproduction pathways of these horticultural plants. Nevertheless, only a small number of ideal aneuploid materials have been excavated in horticultural plants, especially in fruit trees [15–18].

To efficiently breed excellent plant germplasms by aneuploids, it is necessary to study the chromosomal karyotype changes that are rapidly generated during triploid sexual reproduction. However, the types and degrees of these changes, potential reasons, and their impact on phenotypes have only been reported in the model plant *A. thaliana* [5], but remain to be fully understood in the other plants. There are two main reasons for this: 1) the low fertility of triploids means insufficient progeny can be collected; 2) despite great advances in genomic technology, the means to identify aneuploid karyotypes remain limited. The low fertility of triploid is related to the plant’s tolerance to aneuploidy. Some genes regulating aneuploid growth and development have been reported in humans [19] and yeast [20], but there were no relevant reports in plants. Moreover, traditional cytological karyotype identification remains challenging for plants with numerous chromosomes, small chromosomes, or chromosomes of similar shape and size [21]. Chromosome *in situ* hybridization is cumbersome, time-consuming, expensive, and requires extensive operator experience [ 22, 23]. Quantitative fluorescent-polymerase chain reaction (QF-PCR) and simple-sequence repeat-quantitative polymerase chain reaction (SSR-qPCR) require the development of new molecular markers [24, 25]. DNA sequencing (i.e. single-nucleotide polymorphism (SNP) arrays and whole-genome sequencing) is expensive [11, 26].

Loquat is a subtropical fruit tree native to China. *Eriobotrya japonica* (2n = 2x = 34) is the only *Eriobotrya* that is a cultivated species on fruit trees [27]. Hence, the genetic background of cultivated loquat is relatively narrow. For a long time, thousands of triploids have been selected among the progeny of diploid loquats [28]. Most triploid loquats have low fertility, and some triploids are bred into seedless varieties [28–31]. Surprisingly, the triploid loquat line Q24 exhibited female fertility, and most of the progeny of the line were identified as aneuploid under open pollination conditions [9]. These aneuploids provide ideal materials for breeding some novel loquat germplasm by aneuploids. Wen *et al.* [25] developed a rapid and low-cost method for identifying the karyotype of aneuploid loquat. The method makes it possible to identify large-scale aneuploid karyotypes in a short time at a low cost. However, the SSR-qPCR markers were based on linkage maps of apple and pear, and the accuracy of this method in identifying aneuploid loquat karyotypes was 62.5%~88.9%. Until now, several chromosome-level loquat genomes have been published [32, 33]. These data provide an opportunity to reconstruct more accurate SSR-qPCR methods at the chromosome level to identify loquat karyotypes.

In this study, we employed three diploids with diverse genetic distances as male parents to cross-pollinate the female fertile triploid loquat Q24 to construct three experimental populations. Chromosome numbers and karyotypes of triploid loquat Q24 offspring were confirmed using chromosome observation and the improved SSR-qPCR method. On this basis, the genetic characteristics of triploid loquat chromosomes were revealed through large-scale population karyotype analysis. Then, the segregation distortion phenomenon of heterozygous alleles of Q24 in the cross-offspring population was found by whole-genome resequencing. Allelic genes *EjRUN1–1* and *EjRUN1–2*, which functioned in enhancing triploid fertility, were selected and identified in a segregation distortion region. Furthermore, we provided detailed research on the impact of chromosome number and dosage variation on the phenotypic consequences of aneuploid loquat. This study was the first to systematically reveal the perennial triploid woody plant’s genetic characteristics and underlying reasons. These results make it possible to use aneuploid in loquat germplasm resource innovation and provide a crucial reference for profoundly understanding the fertility and genetic characteristics of triploid plants.

## ‌Results

### The chromosome number of the triploid loquat Q24 offspring deviated from the theoretical value distribution

**Figure 1:**
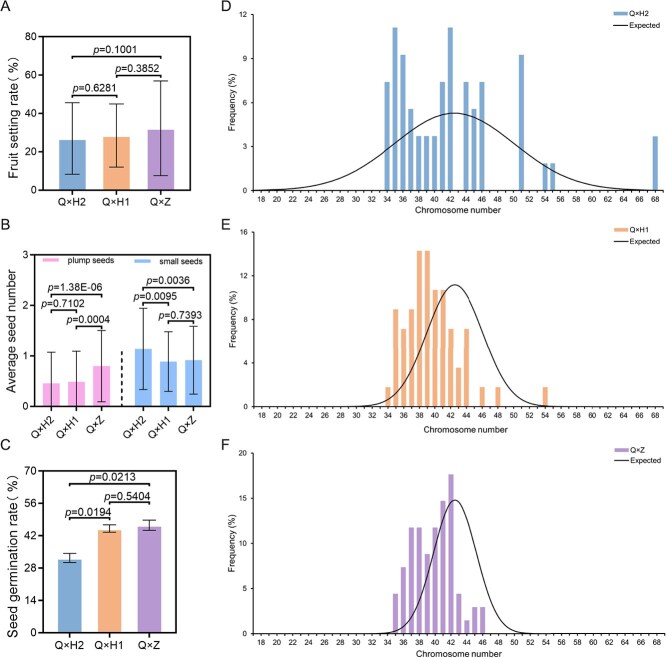
Fruit setting, seed number, and offspring chromosome number distribution of three different cross-combinations. **A**, Fruit setting rate. **B**, Average seed number per fruit. **C**, Seed germination rate. **D**, Distribution of chromosome numbers in F1 progeny of Q24 × Huabai 2. **E**, Distribution of chromosome numbers in F1 progeny of Q24 × Huabai 1. **F**, Distribution of chromosome numbers in F1 progeny of Q24 × Zaozhong 6. The column diagram indicates the actual distribution of chromosome numbers. The curved line indicates the expected distribution of the chromosome numbers (normal distribution based on random chromosome segregation) in the offspring of the triploid loquat and diploid loquat hybrids, with 42.5 as the mean value and a variance equal to the actual distribution. The variances corresponding to D–F are 7.57, 3.57, and 2.69, respectively. The *y*-axis represents the frequencies of the loquat plants with different chromosome numbers. HB1 indicates ‘Huabai 1’; HB2 indicates ‘Huabai 2’; ZZ indicates ‘Zaozhong 6’.

To assess the distribution pattern of chromosome numbers in triploid loquat offspring, we used three diploids with diverse genetic distances (Fig. S1) as male parents to cross-pollinate the female fertile triploid loquat Q24. The genetic distance between Q24 and ‘Huabai 2’, ‘Huabai 1’, and ‘Zaozhong 6’ were 0.179, 0.227, and 0.382. The average fruit setting rates of Q24 × ‘Huabai 2’ (Q × H2), Q24 × ‘Huabai 1’ (Q × H1), and Q24 × ‘Zaozhong 1’ (Q × Z) were, respectively, 26.96%, 28.51%, and 32.28% (Fig. 1A). The average number of plump seeds for Q × H2, Q × H1, and Q × Z were, respectively, 0.46, 0.49, and 0.80, and there was a significant difference between Q × Z and the other two combinations (Fig. 1B). The germination rates of plump seeds of Q × H2, Q × H1, and Q × Z were 32.39%, 45.15%, and 46.57% (Fig. 1C). It can be seen that the higher the genetic distance between parents, the higher the fruit setting rate, the average seed number, and the seed germination rate of their hybrid combination. Finally, 54, 56, and 68 loquat strains were respectively obtained from the F1 generations of Q × H2, Q × H1, and Q × Z.

Next, the number of chromosomes in each plant was confirmed according to microscopic observation of mitotic chromosomes. There were no loquat plants with <34 chromosomes found in the three F1 populations, and the chromosome numbers of plants in the three F1 populations were mainly distributed between 34 and 51 (92.59%, 98.21%, and 100% of Q × H2, Q × H1, and Q × Z, respectively) (Fig. S2, Fig. 1D–F). The distribution of chromosome numbers in the three F1 populations deviated from the theoretical value distribution. Specifically, the distribution of chromosome numbers in the F1 population of Q × H2 did not form a main peak. The main peaks of chromosome numbers in the F1 population of Q × H1 were 38 and 39, and the main peak in F1 population of Q × Z was 42 (Fig. 1D–F). The results also showed that the greater the genetic distance between the female and male parents, the more pronounced the distribution of chromosome numbers in the F1 progeny tended to be between 34 and 42 (Fig. 1D–F, Fig. S1). In addition, for the three F1 populations as a whole, the frequency of chromosome numbers ranging from 34 to 42 exceeded the theoretical distribution. In contrast, the frequency of chromosome numbers of 42 to 50 was far below the theoretical distribution. The number of chromosomes (93.82%) ranged from 34 to 46, peaking at 42 with a frequency of 12.36% (Fig. S3). This skewed distribution indicated that the chromosome numbers in the cross-offspring of triploid loquat Q24 experienced selection.

### The cross-progeny of triploid loquat forms a swarm of aneuploids with extensive karyotype variation

**Figure 2:**
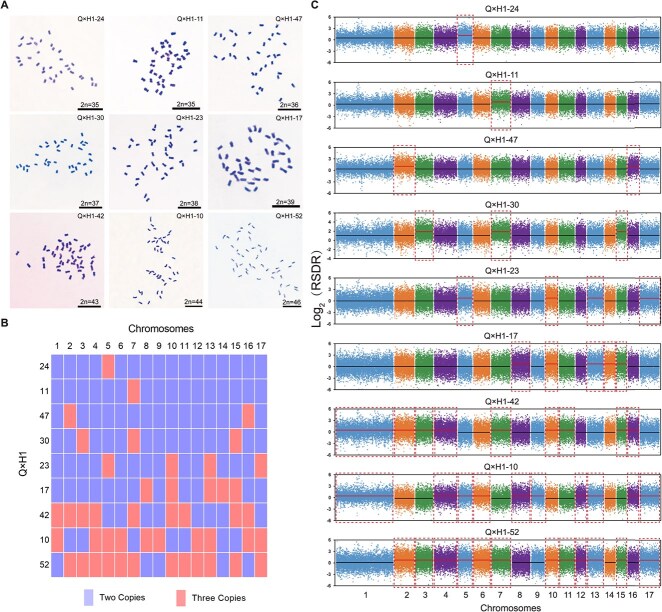
Verification of the improvement SSR-qPCR method for identifying aneuploid loquat karyotypes. **A**, Mitotic metaphase chromosomes of nine aneuploids from Q × H1 cross. The labels of plants were in the upper right corner of the chromosome photograph, and the chromosome numbers are showed in the lower right corner above the bars. Bars = 10 μm. **B**, Molecular karyotype of the nine aneuploid loquat strains identified by the improved SSR-qPCR method. The molecular karyotype pattern of aneuploid loquat was constructed. The plant labels are shown on the left of the block diagram. Every block indicates one chromosome, and chromosome numbers are noted on the upper side. **C**, The ddRAD-seq was used for molecular karyotype identification of aneuploid loquats. Each dot shows the log_2_(RSDR) value of every SNP locus on chromosomes. SNPs were sorted according to their position on the chromosome, at equidistance. The plant labels are noted on the top of every scatter diagram, and the chromosome No. appears on the bottom of these scatter diagrams. The black and red horizontal solid lines represent two and three chromosome copy numbers, respectively. The chromosome numbers of plants were counted as: 2 × black horizontal solid lines +3 × numbers of red horizontal solid lines.

Naturally, to confirm the accuracy of these new markers in identifying the karyotype of triploid loquat Q24 hybrid offspring, data from double digest restriction-site associated sequencing (ddRAD-seq) and chromosome numbers were used to validate the improved SSR-qPCR method. Nine loquat samples were randomly selected from each hybrid as experimental materials. The results showed that the number of chromosomal abnormalities of all materials identified by the improved SSR-qPCR method was consistent with the results obtained by microscopic observation of mitotic chromosomes (Fig. 2A and B; Figs S5A and B and S6A and B; Data S2). The ddRAD-seq results were consistent with the molecular karyotypes detected by the improved SSR-qPCR method (Fig. 2C; Figs S5C and S6C). Therefore, the improved SSR-qPCR method can unambiguously identify the karyotypes of hybrid offspring with triploid loquat Q24 as the female parent and diploid loquat (‘Huabai 1’, ‘Huabai 2’, and ‘Zaozhong 6’) as the male parent. In addition, we also tested the applicability of this method in different loquat varieties. The results indicated that the improved SSR-qPCR method can be applied to identify the aneuploid karyotypes of different triploid cultivated loquat offspring (Fig. S7, Data S3).

To unambiguously identify the karyotype of aneuploid loquat, the SSR-qPCR method was improved. The SSR molecular markers on each chromosome were developed based on the ‘Seventh Star’ loquat genome (Data S1). According to PCR and agarose gel electrophoresis, 105 nonpolymorphic SSR molecular markers were selected from 637 SSR molecular markers (Fig. S4). After qPCR detection, 21 SSR molecular markers were selected from 105 SSR molecular markers to obtain stable ΔRn values in Q24, ‘Huabai 1’, ‘Huabai 2’, and ‘Zaozhong 6’ (Tables S1–S2). The 21 specific SSR molecular markers covered all 17 loquat chromosomes. Taken together, we obtained homozygous allelic markers with stable ΔRn values on each chromosome of four parental materials.

**Figure 3:**
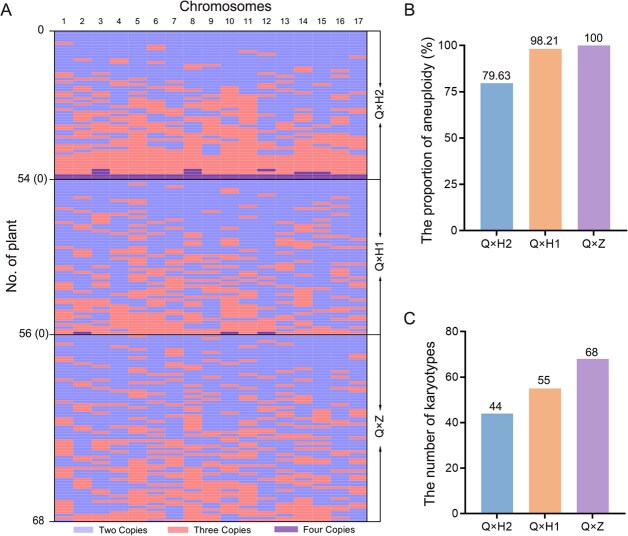
Chromosome copy number variation of hybrid progenies of triploid loquat Q24. **A**, Based on the improved SSR-qPCR results, molecular karyotype heatmaps were constructed for each loquat plant in three hybrid populations. Different colors represent chromosome copy numbers from 2 to 4, and all were labeled on the bottom of the heatmaps. The numbers above the heatmap represent 17 chromosomes of loquat. Different hybrid crosses are displayed on the right. **B**, The proportion of aneuploid loquat plants in different hybrid populations. **C**, Karyotype numbers in different hybrid populations.

Then, we further interrogated the chromosome karyotypes of F1 populations using the improved SSR-qPCR method. The plants of three F1 populations were considered as a whole, and 178 loquat plants had 163 molecular karyotypes, of which 166 aneuploid plants had 160 molecular karyotypes (Fig. 3A). The results indicated that triploid loquat Q24 can produce aneuploid progenies with extensive karyotype variation. Forty-four molecular karyotypes were detected from 54 loquat plants in the F1 progeny of Q × H2. Among them, 11 loquat plants were euploid, with four diploid plants, five triploid plants, and two tetraploid plants. Forty-three loquat plants were aneuploid, accounting for 79.63% of the total number of F1 progeny in Q × H2, and 41 molecular karyotypes were detected (Fig. 3B and C). In the F1 population of Q × H1, 55 molecular karyotypes were detected in 56 loquat plants, of which only one diploid loquat plant was found. Fifty-five aneuploid plants (98.21% of F1 progeny with Q × H1) were detected 54 molecular karyotypes (Fig. 3B and C). Notably, in the F1 population of Q × Z, all 68 plants were aneuploids, each exhibiting a unique molecular karyotype (Fig. 3B and C). The results demonstrated that as the genetic distance increased between the triploid loquat Q24 and the male plants, the rate of aneuploidy in the F1 progeny and the diversity of their molecular karyotypes also increased.

### The function of genes on chromosomes and the number of interchromosome collinear gene links constrained the transmission frequency of Q24 chromosome

**Figure 4:**
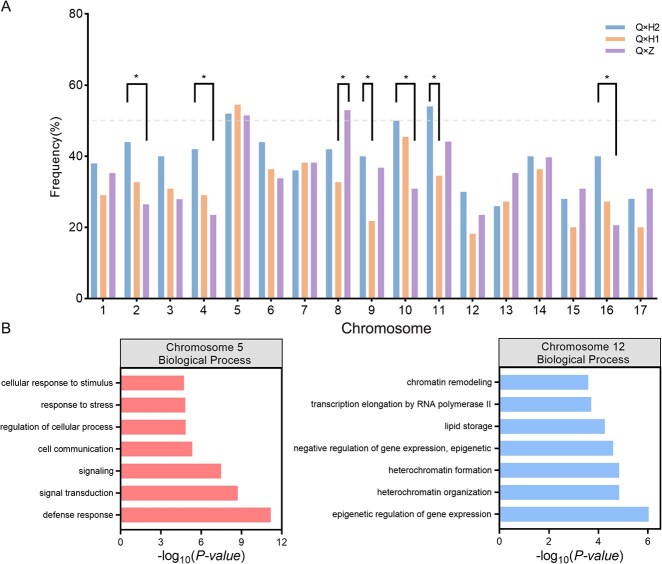
Aneuploidization tendency of Q24 different chromosomes. **A**, The frequency at which each chromosome of Q24 as the disomy was transmitted to offspring plants in three cross-combinations. All three aneuploid populations were shown in different colors. The differences between different cross types are compared using a proportion test. The gray dotted line represents a value of 50%. **B**, GO enrichment analysis of gene functions on chromosome 5 and chromosome 12 of loquat.

Based on large-scale population molecular karyotypes, the genetic characteristics of triploid loquat chromosomes were studied by comparing the frequency of each chromosome of Q24 as two chromosome copies (disomy) are being transmitted to offspring. For three types of crosses, the proportion of most of chromosomes Q24’s being transmitted to offspring as disomy was <50% (Fig. 4A). Chromosome 5 exhibited the highest rate of disomy from the triploid Q24 in F1 populations, and the rates were 52.00% for Q × H2, 54.55% for Q × H1, and 51.47% for Q × Z. In contrast, chromosome 12 exhibited the lowest rate of disomy from the triploid parents in the F1 population, with rates of 30.00% for Q × H2, 18.19% for Q × H1, and 23.53% for Q × Z. We also found that as the genetic distance between Q24 and the father increased, the frequency of chromosomes 2, 4, 10, and 16 of Q24 being transmitted to offspring as disomy decreased (Fig. 4A). Birchler [34] reported that some genes are susceptible to dosage changes and that the overexpression of protein-coding genes can lead to organismal disorders and death. Therefore, this difference may be related to the function of genes on chromosomes. Remarkably, further analysis of gene functions on chromosomes using gene ontology (GO) enrichment, it was discovered that the gene functions on chromosome 5 in biological processes was predominantly enriched in stress response and signal transduction (Fig. 4B). However, the gene functions on chromosome 12 in biological processes were primarily involved related to gene expression and protein synthesis (Fig. 4B). These results indicated that gene functions on loquat chromosomes can affect the frequency of Q24 as disomy transmitted to offspring.

Subsequently, Spearman's rank correlation coefficient was used to investigate whether there was a cooperation or repulsion effect between the nonhomologous chromosome of Q24 as disomy when transmitted to offspring. Specifically, in Q × H2 population, 29 chromosome combinations showed significant positive correlations, of which 18 had moderate positive correlations (Fig. S8A). In the Q × H1 population, 11 chromosome combinations were significantly positively correlated (Fig. S8B). In the Q × Z population, significant positive correlations were noted between 12 chromosome combinations (Fig. S8C). Four chromosome combinations were significantly negatively correlated. Without considering hybridization combinations, 36 chromosome combinations showed significant positive correlations (Fig. S8D). Only one chromosome combination 1 and 2 (Spearman correlation coefficient *r_h_* = −0.169, *P* = 0.031) had a significant negative correlation. These results indicated that some nonhomologous chromosomes of Q24 exhibit varying extents of coordination (positive correlation) or repulsion (negative correlation) when they are transmitted to offspring as disomy.

Further, when combining with the number of collinear gene connections between chromosomes in the ‘Seventh Star’ loquat genome [32], it was found that in the moderately positively correlated chromosome combinations, except for chromosomes 5 and 10, the number of interchromosome collinear gene links between other nonhomologous chromosomes was relatively small. Moreover, there is a negative correlation between chromosomes with a high number of interchromosome collinear gene links. For example, a significant negative correlation was noted between chromosomes 1 and 2 in the Q × Z cross. These results indicated that nonhomologous chromosomes of Q24 as disomy were influenced by the number of interchromosome collinear gene links when simultaneously transmitted to offspring.

### Overexpression of *EjRUN1–1* and *EjRUN1–2* promotes seed vitality of triploid *A. thaliana*

**Figure 5:**
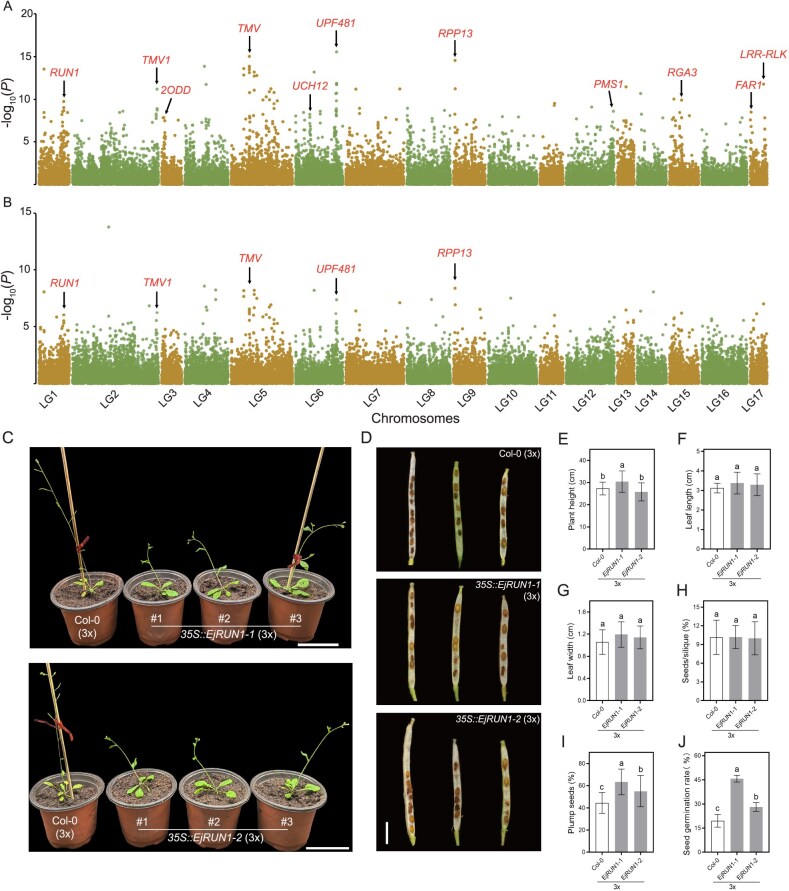
Exploration of distortedly segregated allelic genes. **A**, −Log_10_(*P*) values of all exonic loci of Q24 in pooled genomic DNA of Q × Z aneuploid population based on Law of Segregation and Independent Assortment when Q24 contributed one allelic locus. **B**, −Log_10_(*P*) values of all exonic loci of Q24 in pooled genomic DNA of Q × Z aneuploid population based on Law of Segregation and Independent Assortment when Q24 contributed two allelic loci. Distortedly segregated genes that were related to stress resistance and cell cycle regulators were labeled as red. **C–J**, Phenotypes of *EjRUN1–1* and *EjRUN1–2* overexpressed triploid *A. thaliana* strains. **C**, Overexpression of *EjRUN1–1* and *EjRUN1–2* delayed triploid *A. thaliana* flowering. **D**, Seeds in siliques of overexpressed and wild triploid *A. thaliana* strains. **E**, Plant height. **F**, Leaf length. **G**, Leaf width. **H**, Average number of seeds. **I**, Number of plump seeds. **J**, Seed germination rate. Error bars indicate means ± SEs.

The chromosome numbers and karyotypes in the cross-offspring of triploid loquat Q24 underwent selection. Conceivably, aneuploidy is harmful to cells, but it’s also a choice. Gametes or zygotes that tolerate aneuploidy are selected to form viable plants. We supposed that some aneuploidy tolerance genes in the Q24 genome are passed on to the offspring so that the plants can enhance the tolerance to aneuploidy. This hypothesis can be determined by genome resequencing to detect the enrichment degree of allelic loci in aneuploid plants. Fifty aneuploid plants from Q × Z were used as materials as a pool. Q24 and ‘Zaozhong 6’ were used as contrasts. The frequencies of Q24 allelic loci in the aneuploid pool were tested based on the Law of Segregation and the Law of Independent Assortment (Fig. S9). A *chi*-square test was used to examine the exon allelic SNPs loci with extreme segregation distortion in the aneuploid pool. In theory, if log_10_(*P*) values were >2.00, segregation distortion would be regarded as occurring. The results showed that there were many loci with log_10_(*P*) values >2.00, and the genetic inheritance of triploid loquat Q24 exhibits the characteristics of segregation distortion (Fig. 5A and B). Annotations of genes in the extremely distorted segregation regions (i.e. loci with log_10_(*P*) values >5.00) indicated that these genes were mainly related to stress resistance and cell cycle regulators, such as *RUN1*, *TMV*, *2ODD*, *RPP13*, and *PMS1*.

To validate the function of these genes with segregation distortion, two homolog genes of a disease resistance gene, *EjRUN1*, which were named *EjRUN1–1* and *EjRUN1–2*, were isolated from triploid loquat Q24. *EjRUN1–1* has 35 bases deletions in the coding exons compared to the reference genome (Seventh Star loquat genome). Meanwhile, *EjRUN1–2* exhibits 14 SNPs and 18 bases deletions in the coding exons (Fig. S10A). To confirm the function of *EjRUN1–1* and *EjRUN1–2*, a plant binary expression vector originating from pCAMBIA2300 was constructed for plant genetic transformation in wild-type *A. thaliana*. A total of five homozygous overexpression strains of T3 generation were obtained by resistance and PCR screening (Fig. S10B and D). All the genetically modified strains were late-flowering phenotype (Fig. S10E). The plant height (Fig. S10G), leaf size (Fig. S10H and I), seed number (Fig. S10F, J, and K), and seed germination rate (Fig. S10L) of the overexpressed strains had no significant differences from those of the nontransgenic wild-type plants.

Thus, we used the diploid wild-type and homozygous overexpressed strains as male parents to cross-pollinate the tetraploid wild-type *A. thaliana*. Five triploid transgenic strains were obtained by flow cytometry and PCR experiment (Fig. S11A and B). Overexpression of *EjRUN1–1* and *EjRUN1–2* delayed flowering of triploid *A. thaliana* (Fig. 5C). The plant height of triploid overexpression strains *EjRUN1–1* was markedly higher than those of the nontransgenic triploid wild-type plants (Fig. 5E). However, there was weak significant difference in leaf size and average number of seeds between transgenic plants and nontransgenic plants (Fig. 5D, F, and H). The plump seeds and seed germination rates showed that transgenic triploid strains were severely higher than those of nontransgenic triploid wild type (Fig. 5I and J). These findings confirmed that the distorted segregation genes *EjRUN1–1* and *EjRUN1–2*, produced by the triploid loquat Q24, enhanced the seed viability of triploid *A. thaliana*. Furthermore, the effect of *EjRUN1–1* was more pronounced than that of *EjRUN1–2*.

### The effects of chromosomal number and dosage variation on aneuploid phenotypes

The dosage of aneuploidy chromosomes was imbalanced and changed the copy number of many genes, thereby affecting the phenotype of the organism (Fig. S12). Here, we measured four morphological traits related to plant growth and development, namely plant height, trunk diameter, leaf length, and leaf width, using 1.5-year-old seedlings (21 from Q × H1 hybrid offspring, 54 from Q × H2 hybrid offspring, and 54 from Q × Z hybrid offspring) as experimental materials (Data S4). The phenotypic consequences of aneuploidy in the diverse types were analyzed from three aspects at the population level.

**Figure 6:**
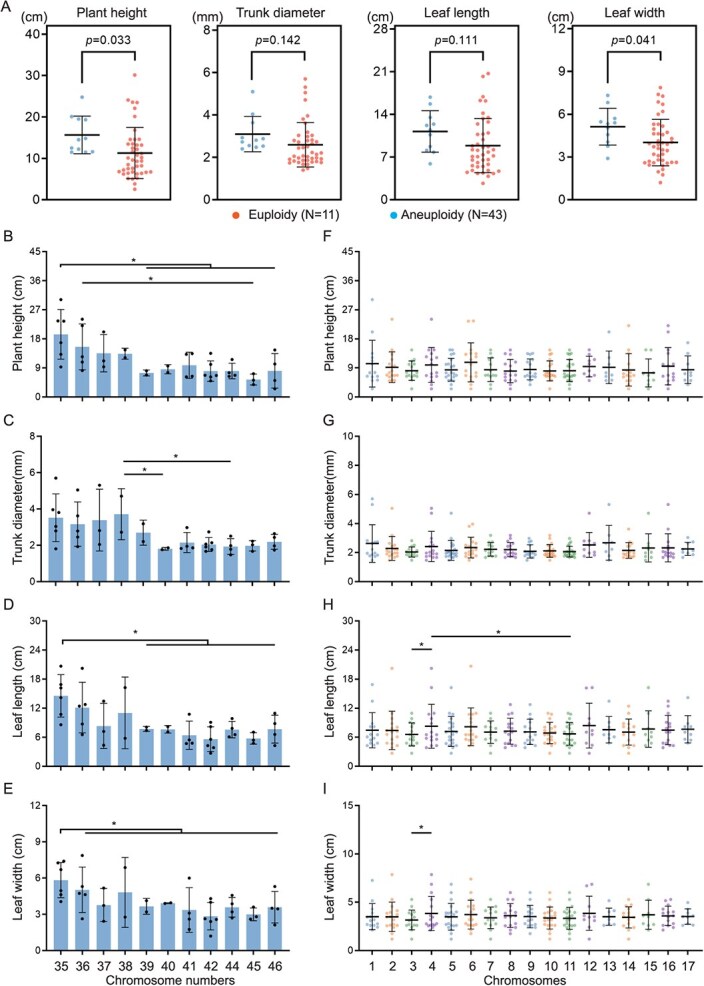
The impact of chromosome number and dosage variation on the loquat phenotypes. **A**, Comparison of the four phenotypes between the aneuploid group and euploid group in F1 population of the Q × H2 cross-combination. Each point represents a hybrid plant. The data on the scatter plots represent the *P*-value of the Student's *t*-test. **B**–**E**, The effects of chromosome number variation on the plant height, trunk diameter, leaf length, and leaf width of Q × H2 offspring, respectively. The *x*-axis represents the chromosome numbers of loquat strains. **F–I**, The effects of chromosome dosage changes on the plant height, trunk diameter, leaf length, and leaf width of Q × H2 cross-offspring, respectively. The *x*-axis represents the 17 chromosomes of loquat. Each dot represents a loquat plant. One-way ANOVA: ^*^, *P* <0 .05.

We compared the phenotypic differences between aneuploid loquat and euploid loquat. The F1 progeny of Q × H2 were selected because there were 11 euploids and 43 aneuploids in this hybrid under the same genetic background. Thus, the materials were divided into two groups, the euploid group and the aneuploid group, and the differences in the four phenotypic traits between the two groups were evaluated using Student's *t*-test. The results showed that the average values of plant height, trunk diameter, leaf length, and leaf width in the euploid group were greater than those in the aneuploid group. The plant height and leaf width in the euploid group were significantly greater than those in the aneuploid group (Student's *t*-test, *P* =0 .033, *P* =0 .041) (Fig. 6A). The coefficients of variation for plant height, trunk diameter, leaf length, and leaf width in the aneuploid group were 54.88%, 40.37%, 50.03%, and 40.61%, respectively, whereas the coefficients of variation for plant height, trunk diameter, leaf length, and leaf width in the euploid group were 28.98%, 26.92%, 30.13%, and 25.13%, respectively (Table S3). It is evident that the aneuploid loquat group exhibited more significant phenotypic variation in plant height, trunk diameter, leaf length, and leaf width than the euploid loquat group.

We further investigated the effect of different chromosome numbers on the phenotype of aneuploid loquats. For this purpose, aneuploid strains with the same number of chromosomes were divided into a group, and the differences in four phenotypic traits were compared using one-way ANOVA. In the F1 progeny of Q × H2, decreasing trends in plant height, trunk diameter, leaf length, and leaf width were noted as the number of chromosomes increased (Fig. 6B–E). The genomic imbalance was most severe among the plant phenotypes when the chromosome number was 2n = 42 or 45. When the chromosome number was 2n = 35 or 36, aneuploidy had a weaker impact on the plant phenotypes of height, leaf length, and leaf width. A similar trend was noted in the Q × Z hybrid population (Fig. S14A–D). When the number of chromosomes was 2n = 40 or 43, the plant phenotypes were strongly affected by genomic imbalance. When the number of chromosomes was 2n = 36, aneuploidy had a weaker impact on the plant phenotypes. However, phenotypic decline with increasing chromosome number was not observed in the Q × H1 hybrid population, which may be related to the insufficient number of progenies in this group (Fig. S13A–D).

We also analyzed the impact of chromosome dosage changes on aneuploid phenotypes. To address this question, aneuploids with the same trisomy were divided into a group, and the effect of increasing the number of each chromosome on the aneuploid phenotype was compared using one-way ANOVA. In the F1 progeny of Q × H2, an increase in the dose of chromosome 4 led to the enlargement of the length and width of the leaves, whereas the dosage of chromosome 3 multiplication causes a narrower leaf phenotype (Fig. 6H and I). In the F1 progeny of Q × H1, increasing the dose of chromosome 2 increased the plant height (Fig. S13E). An increase in the dose of chromosome 8 led to plant dwarfing, a thinner trunk diameter, and a narrower leaf phenotype (Fig. S13E–H). An increase in the dose of chromosome 15 caused an enlarged leaf phenotype (Fig. S13H). In the F1 progeny of Q × Z, plants with an increased dose of chromosome 16 had the greatest average plant height, whereas those with an increased dose of chromosome 4 had the lowest average plant height (Fig. S14E). Plants with increased doses of chromosome 7 had the thickest average trunk diameter (Fig. S14F). An increase in the dose of chromosome 2 increased the length and width of the plant leaves (Fig. S14G and H). An increase in the dosage of chromosome 6 caused a narrower leaf phenotype (Fig. S14H). Remarkably, for the same traits of different cross types, the chromosomal-specific phenotype of aneuploidy exhibited different chromosomal dosage changes.

Collectively, we observed abundant phenotypic variations in aneuploidy compared to euploidy in the triploid loquat Q24 offspring. Moreover, chromosome number and dosage variation affect the phenotypic variation of aneuploid loquat.

## Discussion

### S‌SR-qPCR is a universal method for identifying the aneuploid karyotypes

Loquat chromosomes are a large number (2n = 2x = 34) and relatively small (34~48 Mb), so it is difficult to identify individual chromosomes using chromosome morphological observation and costly by high-throughput sequencing techniques [11, 26, 28, 32]. SSR-qPCR method was more rapid and low cost. The improved SSR-qPCR method can accurately identify the karyotypes of aneuploid progenies of triploid cultivated loquat, because molecular markers of the improved SSR-qPCR method were derived from the chromosome-level genome of loquat. In addition, the SSR-qPCR method exhibits universality. This method can be used to identify the aneuploid karyotype of other plants, even all eukaryotes, as long as chromosome-specific nonpolymorphic molecular markers are developed.

### Inheritance pattern of triploid loquat chromosome numbers

There were about one-third diploids found in *A. thaliana* populations of 3x × 2x and 2x × 3x. Triploid *A. thaliana* produces 8%–10% of haploid microspores [35, 36]. However, the expected proportion of haploid gametes produced by triploid loquat Q24 was only 1.90%. Many aneuploid individuals produced by triploid sexual reproduction cannot develop into viable plants for aneuploidy toxicity, but diploids can survive. Therefore, compared to triploid *A. thaliana*, the triploid loquat Q24 had a stronger tolerance to aneuploidy toxicity. We found that the genetic distance between the parents affected the chromosome numbers and karyotypes of the surviving triploid loquat progenies. As the genetic distance between parents increases, the deviation of chromosome number distribution of the F1 populations of the 3x × 2x from the theoretical distribution becomes more pronounced. This feature was also reflected in the fruit setting rate, average seed number, and seed germination rate. For high-level heterozygosity of the loquat genome, greater genetic distance between parents may buffer the adverse effects of genome imbalance on the organism.

In addition, we did not find any plants with fewer chromosomes than diploids among the surviving offspring of the triploid loquat plants. This finding suggested that reducing one or more chromosome numbers in the diploid background was much more harmful to the organism than increasing the number of chromosomes. Meanwhile, the chromosome numbers in the offspring of the 3x × 2x loquat cross were mainly distributed in the range of 34~46, which is close to that of diploids. Cao *et al.* [2] suggested that genome balance and dosage effects drive the transformation of chromosome number in the progeny of allotriploid AAC *Brassica* toward tetraploidy. However, in the case of 3x × 2x loquat cross, whether the distribution of loquat chromosome numbers near diploids was driven by genomic balance and dosage effects requires further investigation.

### Chromosomal genetic characteristics of triploid loquat

The hybrid progenies of triploid loquat form a swarm of aneuploids with extensive karyotype variation. The chromosome base of loquat is 17 [32, 33]. For the 3x × 2x cross, if one or two copies of each chromosome in the individual are obtained from the triploid parent, then the offspring will theoretically form 131 072 karyotypes (2^17^ [17], of which two are diploid and triploid). Our results showed that 178 loquat plants of the 3x × 2x crosses’ offspring had 163 molecular karyotypes. This may be related to the small number of plants in the hybrid progeny population. Triploid *A. thaliana* can theoretically produce 32 types of gametic karyotypes. However, more aneuploid plants with *A. thaliana* trisomy (2n + 1) were detected than those with other karyotypes in their hybrid offspring [36]. Maybe the *A. thaliana* genome of aneuploids with one more chromosome than diploid was more stable than other aneuploids. Nevertheless, aneuploid plants with loquat trisomy (2n + 1) were rarely detected in the hybrid offspring of triploid loquat. This may be related to the numerous chromosomes and complex genome of loquat. In addition, like other triploid plants, triploid loquat offspring can directly produce tetraploid loquat strains, indicating that triploids can directly promote the formation of polyploids.

All chromosomes of the triploid loquat Q24 could be transmitted as the disomy but in different frequencies. Different chromosome disomic transmission efficiencies differed in many other plant species [36]. Our research found that the gene functions on chromosomes can affect the transmission frequency of Q24’s chromosome. For example, the genes related to biotic and abiotic stress may play a role in regulating aneuploidy tolerance in loquat, thus minimizing the detrimental effects of aneuploidy on the organism [37]. In our study, the genes on chromosome 5 primarily relate to adaptation to biotic and abiotic stress. This could be the reason why Q24’s chromosome 5 as the disomy was transmitted to offspring with the highest frequency in different loquat cross-populations. Birchler [34] reported that some genes are susceptible to dosage changes and that the overexpression of protein-coding genes can lead to organismal disorders and death. The functions of the genes located on chromosome 12 of Q24 were primarily associated with the regulation of protein synthesis. Having an additional copy of chromosome 12 can disrupt the stoichiometry of related protein complexes, resulting in significant adverse effects on the plant. As a result, chromosome 12 of triploid loquat Q24 as the disomy was transmitted to offspring with the lowest frequency.

Another significant finding is that the triploid loquat Q24 produces some specific chromosome combinations that are easily transmitted to offspring simultaneously as the disomy. These specific chromosome combinations were related to the number of interchromosome collinear gene links. This may be due to the lesser number of interchromosome collinear gene links, which can avoid gene redundancy and buffer the impact of genome imbalance and thus ensure the survival of aneuploid plants. Conversely, the closer number of interchromosome collinear gene links might exacerbate the harmful consequences of genomic imbalance on cells. Therefore, it is challenging to detect simultaneously in triploid offspring.

This study is the first to demonstrate the chromosome inheritance patterns of triploid plants that possess numerous chromosomes and experience resent polyploidization.

### Possible molecular mechanism of Q24 tolerance to aneuploidy

Aneuploidy was a severe stress on cells. To maintain the life activities of cells, the expression of certain tolerance-related genes for aneuploidy will be induced. For example, in PUF60, a cell cycle regulator, heterozygous mutations lead to developmental genetic disease, while elevated copy number and/or expression in various cancers have potential oncogenic effects [38]. A homologous mutation of *hPMS1* in human cells can lead to increased cancer [39]. It was found that some distortedly segregated loci were found in the aneuploid population, and some genes related to stress resistance and cell cycle regulation were found in these segregation distortion regions. We demonstrated through transgenic experiments that *EjRUN1–1* and *EjRUN1–2* can promote seed viability of triploid *A. thaliana* offspring. This further indicated that some genes involved in regulating aneuploid tolerance in these segregation distortion regions. This provides us with a pathway to explore genes more involved in regulating aneuploid tolerance. This work can be carried out in the near future, and these regulation networks will be uncovered. On this basis, the use of gene editing technology to create more aneuploidy-resistant materials of excellent phenotypes provides a theoretical basis.

### Phenotypic response of loquat plants to aneuploidization

Chromosomal dosage imbalances have serious phenotypic consequences for organisms [40–42]. Nevertheless, plants are more tolerant of aneuploidy than are animals [34, 43]. Two broad categories of phenotypes are associated with aneuploidy: the general phenotypes shared by all or most aneuploidies and the specific phenotypes associated with specific chromosomal dosage changes [ 11, 13, 44, 45]. This study measured four phenotypes that reflect plant growth and development. Our observations indicated that aneuploid loquats exhibit general phenotypes of delayed growth and development compared to euploid loquats. For example, the average plant height, trunk diameter, leaf length, and leaf width of the aneuploid loquat group were lower than those of the euploid loquat group. Sheltzer *et al.* [37] suggested that the general phenotypes of aneuploidy are caused by genomic imbalance, which can induce the misexpression of many genes related to cell division and the cell cycle. Moreover, the aneuploid loquat group exhibited more abundant phenotypic variation than the euploid loquat group, providing more options for cultivating new materials with excellent traits.

In addition, the phenotypes of aneuploid loquat were associated with an increase in the dosage of specific chromosomes. Again, we should caution that specific aneuploid loquat phenotypes may be related to paternal effects. For example, in the Q × H1 cross, the extra chromosome 2 led to an increase in plant height. In the Q × Z cross, the average plant height of the extra adding chromosome 16 was the greatest. Plant height, trunk diameter, leaf length, and leaf width are quantitative traits, which are generally controlled by the accumulation of multiple genes [46–48]. Loquat is a heterozygous plant. It can be imagined that even aneuploids with the same karyotype have different genotypes for many genes on the chromosome. Therefore, this phenomenon may be related to the high heterozygosity of the loquat genome. The effects of specific chromosome dosage changes on plant phenotypes have also been reported in *A. thaliana* [5], rice [11], and wheat [13]. We also found that the plant height, trunk diameter, leaf length, and leaf width of aneuploid loquats decreased with increasing chromosome number. The tendency was similar in Q × H2 and Q × Z hybridizations, and the tendency was not obvious in Q × H1 hybridization for too few aneuploids. These chromosome number effects were not paid attention to in other plants. The loquat genome has undergone two whole-genome replication events, and the collinearity between many interchromosomes is high [32, 33]. The accumulation of genes with the same function may further amplify the harmful effects of genomic imbalance to organisms, so the aneuploid phenotype becomes more severe as the chromosomal number increases. We raised a bold assumption that aneuploidy effects were different on plants with different chromosome numbers, and effects of chromosome dosage and allelic genes might work on this basis. Epigenetics modification plays a major role in aneuploid phenotype response, and some of these modifications can be transmitted to progenies, i.e. the long-term effect of aneuploidy works.

## Materials and methods

### Plant materials

Female fertile triploid loquat line Q24 was used as the female parent and crossed with three diploid loquat varieties (‘Huabai 2’, ‘Huabai 1’, and ‘Zaozhong 6’). Three hybrid populations were constructed: Q24 × Huabai 2 (Q × H2 for short), Q24 × Huabai 1 (Q × H1 for short), and Q24 × Zaozhong 6 (Q × Z for short). Q24 were not fertile when self-pollinated [9]. And pollinated inflorescences were sealed by windtight kraft bags for at least 2 weeks until the styles dried up. Therefore, all seeds developed from pollinated flowers were surely hybrid seeds. All seeds were cultured in mixed substrate (2 V turfy soil +2 V peat soil +1 V river sand). All these plants were planted in the polyploid loquat germplasm nursery of Southwest University (Beibei District, Chongqing, China). *Arabidopsis thaliana* (2x, Col-0) was used for genetic transformation experiments. Tetraploid *A. thaliana* [49] (4x, Col-0) was used to prepare triploid *A. thaliana*. All plant materials were grown in a greenhouse under normal conditions (16 h light/8 h dark) at 22°C.

### Chromosome preparation

Chromosomes at mitotic metaphase were prepared according to the method reported by Dang *et al.* [18]. Root tips of 1.0~1.5 cm in length were taken and treated with 0.002 mol/l 8-hydroxyquinoline aqueous solution for 3 h in the dark. Then, the root tissue was fixed in Carnoy’s fluid (3 V methyl alcohol +1 V acetic acid solution). Enzymatic hydrolysis was performed in a mixed enzyme solution of 3% cellulose and 0.3% pectinase at 37°C for 3 h. Chromosome samples were obtained after spreading root tips on slides. Finally, 5% Giemsa solution was used to stain chromosomes. Chromosomes were observed under a microscope (Olympus, Tokyo, Japan) and photographs were taken by using a charge-coupled device camera.

### DNA extraction and examination

The improved CTAB method was used to isolate loquat genomic DNA [50]. DNA concentration and mass were determined by an ND-1000 NanoDrop spectrophotometer (Eppendorf, Germany). The quality of the DNA was then tested using a 0.8% agarose gel. All genomic DNA were diluted to 50 ng/μl for subsequent experiments.

### Development SSR molecular marker primer for molecular karyotype analysis

The SSR markers for each chromosome of the Seventh Star loquat genome were isolated using Thiel *et al.* [51]. The specific SSR markers on each chromosome were screened in Microsoft Excel (2021). To screen out nonpolymorphic SSR primers on each chromosome, PCR and agarose gel electrophoresis were carried out according to the method reported by Wen *et al.* [25]. Genomic DNA of Q24, ‘Huabai 2’, ‘Huabai 1’, and ‘Zaozhong 6’ were used as template DNA. Only primers that can only amplify a single band were retained and further screened by qPCR.

### qPCR for further primer screening and molecular karyotyping

The qPCR was performed using a one-step real-time quantitative fluorescence PCR instrument (qTOWER3G IVD, Analytikjean). The reaction system was 20 μl, including 2 μl of genomic DNA (50 ng/μl), 0.4 μl each of the forward and reverse primers (0.25 μM), 10 μl of 2 × NovoStart® SYBR qPCR SuperMix Plus (Novoprotein Scientific, Inc.), and an appropriate amount of ddH_2_O to increase the volume to 20 μl. The reaction procedure was as follows: 95°C for 1 min, 40 cycles of 95°C for 20 s and 60°C for 1 min, and then melting curve analysis at 60°C–95°C.

Molecular karyotype analysis of loquat was performed following the method reported by Wen *et al.* [25]. Briefly, qPCR experiments were conducted using euploid loquats (Q24, ‘Huabai 2’, ‘Huabai 1’, and ‘Zaozhong 6’) as materials and nonpolymorphic SSR molecular markers as primers. In the exponential growth phase, three different points were taken to calculate the average value of ΔRn. Using the ΔRn of MK30145 as a control, all the ΔRn values of the SSR molecular markers were standardized (i.e. the ΔRn value of the SSR markers/ΔRn value of the MK30145 markers). SSR molecular markers with stable ΔRn ratios in four euploid materials were selected. At least one marker was selected on each chromosome.

Next, SSR molecular markers with stable ΔRn ratios in four euploid materials were used to perform fluorescence quantitative PCR experiments on individuals of each triploid Q24 hybrid offspring, and the values of each marker were used to calculate the ΔRn ratio. Then, normalization was performed again (corresponding SSR molecular markers, ΔRn ratio of the sample to be tested/ΔRn ratio of the average euploid loquats), and the number of copies of the chromosome was estimated. In theory, when MK30145 was located on unvaried chromosomes, the values were 1 (2x, 3x, and 4x karyotypes), 0.5 (2x − 1 karyotype), 1.5 (2x + 1 karyotype), or 1.3 (3x + 1 karyotype). When MK30145 was on a numerically altered chromosome (extra addition), those values were 0.66 (2x karyotype) or 1 (2x + 1 karyotype).

### Double digest restriction-site associated sequencing and whole-genome resequencing

The extracted genomic DNA was subjected to restriction endonuclease digestion at 37°C for 5 h. Vahtstm DNA clean beads were used to remove large and small segments after enzyme digestion. The adapters were ligated, and the DNA fragments of the adapters were amplified with PCR amplification. Finally, the library fragments were purified by 2% agarose gel electrophoresis. The 220- to 450-bp fragments were inserted into the library. Using next-generation sequencing (NGS) based on the Illumina Nova-Seq sequencing platform, paired-end (PE) sequencing was performed on the 2 × 150 bp library [52]. One library was constructed for each sample. For whole-genome resequencing, the genomic DNA of 50 aneuploidies of Q × Z hybrid offspring were mixed in equal amounts as a pool, and sequenced using the Illumina Nova-Seq sequencing platform. The 2 × 150 bp pair-end method was used for library construction. The genome resequencing data for ‘Q24’ and ‘Zaozhong 6’ were sourced from Wang *et al.* [53].

Fastq (v0.20.0) was used to filter the raw data using the sliding window method. The bwamem (0.7.12-r1039) program was used to compare the filtered high-quality data to the reference genome of seventh star loquat, and the comparison parameters were all set according to the default parameters of bwamem [54]. Picard 1.107 software (http://www.psc.edu/index.php/user-resources/software/picard) was used for sam file sorting and conversion to bam files. The 'FixMateInformation' command was used to ensure consistency of all paired end read information. 'MarkDuplicates' in the Picard software package was used to remove duplicates. The IndelRealigner command in the GATK program was used to realign all reads near Indels to improve the accuracy of SNP prediction. The statistics of the sequence comparison results are shown in Table S4. GATK software was used for SNP detection, and ANNOVAR software was used to annotate SNP loci [55, 56].

### Molecular karyotyping based on ddRAD-seq

To determine the molecular karyotype of aneuploid loquat, we used the relative sequencing depth ratio (RSDR) of the SNPs to determine the loss or gain of chromosomes. Considering that ddRAD-seq yields data with a lower sequencing depth, many false-positive SNPs may be produced, leading to incorrect results. We used RSDR instead of the actual SNP ratio for each SNP [57]. RSDR = sample SNPs sequencing depth/euploid SNPs sequencing depth, with the diploid of Q × H1–5 used as a control. The RSDR was further normalized with log_2_ transformation to facilitate karyotype evaluation. Due to the inconsistent sequencing depth of the samples, through log_2_ transformation, most values were calculated to be approximately 0. If the log_2_RSDR value of each chromosome is constant, the material is considered euploid. If the log_2_RSDR value of each chromosome is not constant, it is aneuploid. If a chromosome is gained, the log_2_RSDR of the chromosome is greater than the constant value. If a chromosome is lost, the log_2_RSDR of the chromosome is less than the constant value.

### Statistical analyses of chromosome inheritance

The effects of different 3x × 2x crosses on chromosomal inheritance were evaluated according to the methods of Henry *et al.* [36]. Briefly, we used loquat strains with chromosome numbers between 34 and 51 from three hybrid offspring as materials. The proportion test was used to compare for each chromosome the percentage of two copy numbers obtained from triploid parents to the total number (the sum of one and two copy chromosomes) in the different cross-combinations. That is, we evaluated whether the 17 chromosomes of the triploid loquat Q24 had equal or different aneuploidy tendencies.

Based on the molecular karyotype of the loquat strain, Spearman rank correlation was performed using IBM SPSS Statistics (version 26) software to evaluate the correlation between the chromosomes when two chromosome copy numbers were obtained from the triploid parent. GraphPad Prism software (version 8.2.1) was used to plot correlation heatmaps between chromosomes.

### Analysis on segregation distortion of aneuploid population

The sequencing depth and genotype information of each SNP locus in pooled DNA of 50 aneuploid F1 hybrids of Q × Z were extracted. Loci with low sequencing depth (generally remove sites <50% average sequencing depth) in the ‘Q24’ and ‘ZaoZhong 6’ were filtered out. Ratios of all allelic SNP loci were calculated, and loci which are heterozygous in Q24 (with 1:3 or 2:3 allelic loci ratios) and homozygous in ‘Zaozhong 6′ were selected. Corresponding allelic loci ratios in pooled DNA were tested by *chi*-square test based on Law of Segregation and Independent Assortment Fig. S9, and *P-*values were recorded. Finally, a scatter plot was drawn with the position of SNP on chromosome as the *x*-axis and the log_10_(*P*) value as the *y*-axis.

### Nucleic acid extraction, gene isolation, sequence analyses, and *A. thaliana* transformation

Total RNA was prepared using the RNA prep pure plant plus kit (No. DP441, Tiangen, China) according to the manufacture’s protocol, and the reverse transcription kit (No. 2641 A, Takara, Japan) was used to synthesize cDNA.

The cDNA of triploid loquat Q24 was used for *EjRUN1–1* and *EjRUN1–2* coding region amplifications with the following primer pairs: F: 5′-ATGCGATGGAAGGGAATAAAGCAG-3′, R: 5′-TCATGGCTTAGAATGGCCGTCGGGA-3′. Multiple sequence alignment was performed using ClustalX and MEGA5 [58].

*EjRUN1–1* and *EjRUN1–2* were cloned into pCAMBIA2300 vectors to construct 3*5 S::EjRUN1–1* and *35 S::EjRUN1–2* vectors for genetic transformation in *A. thaliana*. The overexpressed vectors were transferred to Col-0 *Arabidopsis* by floral dip method [59].

### Phenotypic analysis

Four morphological traits reflecting plant growth and development, including plant height, trunk diameter, leaf length, and leaf width, were measured in the 1.5-year-old seedlings of triploid loquat Q24 hybrid offspring [11]. To investigate the phenotypic effects of different aneuploid loquat strains, the *t*-test and one-way ANOVA were used for statistical analysis.

To determine the traits of *A. thaliana* seeds, each silique was collected in a 1.5 ml tube, and the total number of seeds and the number of plump seeds were counted using a dissecting microscope. Seed germination rates were determined by calculating the number of seedlings for 10 days and comparing it with the total number of seeds sown [36]. The significance of all traits was analyzed by using Student's *t*-test for pairwise comparisons.

## Supplementary Material

Web_Material_uhaf023

## Data Availability

All data referred to are included in the manuscript or are available in supplementary materials.
